# Qualitative study on perceptions of use of Fractional Exhaled Nitric Oxide (FeNO) in asthma reviews

**DOI:** 10.1038/s41533-022-00272-0

**Published:** 2022-03-21

**Authors:** Marta Santillo, Ben Ainsworth, Michelle Helena Van Velthoven, Lucy Yardley, Mike Thomas, Kay Wang, Sarah Tonkin-Crine

**Affiliations:** 1 Nuffield Department of Primary Care Health Sciences, University of Oxford, Radcliffe Observatory Quarter, Woodstock Road, Oxford, OX2 6GG UK; 2 Department of Psychology, Faculty of Humanities and Social Sciences, University of Bath, Bath, UK; 3 Respiratory Biomedical Research Unit, University Hospitals Southampton NHS Foundation Trust, Southampton, UK; 4 Centre for Clinical and Community Applications of Health Psychology, School of Psychology, Faculty of Environmental and Life Sciences, University of Southampton, Southampton, UK; 5 School of Psychological Science, University of Bristol, Bristol, UK; 6 Primary Care, Population Sciences and Medical Education (PPM), University of Southampton, Southampton, UK

**Keywords:** Asthma, Patient education

## Abstract

Current methods to assess asthma and guide inhaled corticosteroid (ICS) dose titration mainly centre on patient-reported symptoms and lung function assessments. However, these methods correlate only weakly with airway inflammation making them unreliable predictors of future exacerbations and ICS requirement. Fractional Exhaled Nitric Oxide (FeNO) is a simple non-invasive objective measure of airways inflammation used predominantly in specialist clinics. Previous qualitative studies have mainly focused on the acceptability of FeNO in secondary care and there is limited insight to support clinicians and patients using FeNO in primary care asthma reviews. This study aimed to explore adult patient with asthma and primary care health care professional (HCP) views on introducing FeNO as part of routine asthma reviews. Twenty-three health care professionals and 22 patients were interviewed over the phone or online. Both groups reported that current asthma reviews are often seen as tick-box exercises and that introducing the FeNO test would make reviews more tailored to the individual patient, rather than relying on subjective patient reports of asthma control. Adults with asthma also highlighted support more open communication and their understanding of asthma, as they desired to feel more engaged in decisions and conversations about their asthma. HCPs reported valuing patient education and empowerment over a paternalistic approach, when time and resources allow. They also recognised FeNO to provide an objective measure of inflammation that could support them in the education and empowerment of patients. FeNO was seen by both groups as a potentially valuable addition to current asthma reviews mainly led by nurses, both for increasing their understanding of current risk of exacerbation and also to provide more tailored and personalised asthma management to patients. Our findings highlighted the need for open and clear communication about how to interpret FeNO results.

## Introduction

Current clinical practice guidelines recommend that asthma patients should be reviewed at least annually to assess symptom control and exacerbation risk, and optimise treatment^1^. Standard methods of monitoring asthma in primary care mainly include patient-reported symptoms and basic lung function assessments, and pharmacologic therapy decisions, such as use of inhaled corticosteroids (ics) and dose adjustments, are based on these assessments. However, these methods are only weakly correlated to airway objective levels of inflammation making them unreliable predictors of future exacerbations. They also may lead to both under-treatment with anti-inflammatory ICS treatment in patients who have inflamed airways but low symptoms (who remain at increased risk of exacerbations) and over-treatment with ICS of patients who continue to report symptoms but who lack objective airways inflammation, and in whom other treatment opinions are more effective^2–4^.

In 2014, the UK National Institute for Health and Care Excellence (NICE) recommended that clinicians should use Fractional Exhaled Nitric Oxide (FeNO)^5^ to diagnose asthma more accurately, and has recommended further research to assess the use of FeNO in monitoring of asthma, where the current evidence of effectiveness in routine care is unclear. FeNO is a simple, non-invasive breath test that provides objective evidence of steroid-responsive eosinophilic airway inflammation^6^. Population-based observational data demonstrate a relationship between fractional exhaled nitric oxide (FeNO) measurements and risk of acute asthma exacerbations^7^. However, FeNO is not currently routinely measured in primary care, where the majority of asthma diagnosis and monitoring takes place^8^.

Although some previous trials comparing FeNO-guided asthma management strategies with symptom- or guideline-driven strategies have suggested that FeNO-guided management may be associated with better clinical outcomes, the evidence base remains equivocal, and the role on FENO in routine primary care asthma management requires further research to clarify. In particular, it is unclear how HCPs and patients should be supported with using FeNO in the context of routine primary care asthma reviews^9^. A study evaluating a decision-support algorithm for asthma management based on FeNO highlighted that protocol deviations from the algorithm occurred in 25% of decisions, indicating that clinicians were frequently over-riding FENO-based recommendations, for reasons that are currently unclear. These findings suggested the importance of understanding uncertainties among clinicians about how FeNO values should be interpreted^10^. A recent qualitative study conducted in secondary care explored the acceptability and feasibility of a FeNO-based management strategy for pregnant women^11^. This study reported that health care professionals wanted appropriate education of both staff and patients on FeNO-based asthma management, and guidance on implementation of FeNO for different settings and models of care.

Previous qualitative work on asthma has explored patient preferences in relation to asthma self-management support^12^, patient understanding of asthma control, and views on communication during routine asthma reviews^13^, but to date there are no qualitative studies of patient views on the adoption or use of FeNO during reviews. Two studies have investigated the acceptability and ease of use of FeNO in nurse-led general practice clinics. Results highlighted that FeNO was seen as an acceptable technique to most patients, both adults, and children^14–16^. A recent qualitative study explored pregnant women’s views on the use of FeNO in antenatal care clinics. Interview participants reported feeling that they had a better understanding of their asthma and felt their asthma was managed more safely and accurately when FeNO was used to guide clinical decision-making. They also felt the FeNO test was quick, easy, and convenient to perform.

This study is part of a wider programme of research to develop and evaluate an online FeNO-guided asthma management intervention in primary care (www.definestudy.com). This research includes Person Based Approach (PBA) intervention development followed by a randomised controlled trial that will evaluate FeNO in primary care routine asthma reviews. The person-based approach (PBA) to intervention development highlights the need to identify barriers and facilitators of the key behaviours, in this case, adoption and appropriate use of FeNO during routine asthma reviews. This study identifies barriers and facilitators to the implementation of FeNO testing in primary care by exploring clinicians’ and adult patients with asthma’s current experience of asthma reviews, asthma management, and their views on adopting and using FeNO testing as part of routine asthma reviews.

## Methods

### Recruitment

This was a qualitative study using semi-structured interviews.

Different strategies were employed to recruit participants. To recruit healthcare professionals, we sought support from Thames Valley and South Midlands Clinical Research Network (CRN) to invite general practices and their staff. To be eligible to participate primary care health care professionals (including general practitioners, nurse practitioners, and pharmacists) had to carry out routine asthma reviews with patients and, where qualified, make decisions about asthma medication. We sought to obtain a maximum variation sample based on job role, years of experience, additional training in respiratory medicine or asthma management, practice list size. Practices expressed their interest in taking part in the study directly to the CRN which then informed the research team by email. Health care professionals eligible to take part were then purposively selected and contacted to arrange an interview.

To recruit adults with asthma, we sent invitations through general practices, advertised through Asthma UK and through Twitter and Facebook. Participants with asthma were invited to contact the research team or let their practice know if they were interested in taking part. To be eligible, people had to be diagnosed with asthma, fluent in English, aged 12 or over, or a parent/carer of a child aged 12-15 diagnosed with asthma. Maximum variation sampling was based on age, gender, time since diagnosis of asthma, and history of previous exacerbations, in order to capture a range of disease severity and control, as we felt these would be key in influencing patients’ perceptions of FeNO. Adults with asthma who expressed interest were contacted by email to arrange an interview.

Before attending the interview, all participants were asked to watch a short video clip of a health care professional showing a patient how to do a FeNO test and explaining the result to him. The video lasted approximately three minutes and was produced by Cogora. It is no longer accessible online at the time of writing. Although Circassia provided funding for the video and checked its content, the focus of the video was instructional rather than promotional. We, therefore, considered it to be suitable clip to show to interview participants, since Covid restrictions meant that it was not possible to produce our own instructional video at the time the interviews were taking place.

### Interviews

Interviews took place over the telephone or online using MS Teams. The interviews had to be carried out remotely because of the COVID-19 pandemic. Interviews were conducted following a semi-structured interview guide. Two topic guides (one for adults with asthma and one for HCPs) were developed by STC and MS. Both included broad open-ended questions and follow up prompts. Topic guides were developed based on existing literature on qualitative studies on patients’ views on asthma reviews, their experience of asthma management, and communication with HCPs^12,13^. Health care professionals were asked about their views and experience of conducting asthma reviews in primary care and about the adoption and use of the FeNO testing as part of routine asthma reviews. Adults with asthma were asked their views and experiences of asthma reviews, and their thoughts about doing a FeNO test as part of their asthma review. All interviews were audio-recorded and transcribed verbatim. Consent was obtained verbally at the start of interviews conducted remotely (i.e., over the telephone or online). The researcher made a written record of this which was stored as for written consent. Participants who took part remotely received a copy of the signed verbal ICF by email. Verbal consent was not audio recorded. The study received NHS REC ethics approval (South Central–Berkshire B Research Ethics Committee, 20/SC/0235).

### Data analysis

Data collection and data analysis occurred concurrently. HCP and patient data were analysed separately. Transcripts were analysed by MS using inductive thematic analysis^17^ on NVivo (version 12)^18^. After reading the transcripts several times to be familiar with the data, MS and STC each coded a subset of the interviews, compared the codes, and discussed any differences and similarities. A coding manual was developed after the first few interviews and was refined during the analytical process. Descriptions of themes and sub-themes were then added. To enhance the trustworthiness of the data the themes were discussed with the wider multidisciplinary team consisting of primary care researchers, psychologists, primary care clinicians, and experts in primary care asthma reviews and FeNO testing.

### Reporting Summary

Further information on research design is available in the Nature Research Reporting Summary linked to this article.

## Results

Twenty-three health care professionals completed an interview (5 GPs, 13 Practice Nurses, and 5 Clinical Pharmacists). Table 1 reports participants’ characteristics. Participants had no or little prior knowledge or experience of measuring FeNO in primary care with only 3 HCPs having had any experience of using the test in the past (1 HCP used FeNO in a respiratory hub and 2 HCPs had experience of using a FeNO analyser during training events). Twenty-two adults with asthma completed an interview. Of these, eight had experience of doing a FeNO test (five in secondary care and three in primary care). All interviews were conducted by one experienced primary care qualitative researcher (MS) between July 2020 and February 2021. Interviews lasted between 25 and 45 min.

**Table 1:** Summary of HCP and patient characteristics.

	HCPs	Patients
Age (mean, range)	n/a	39, 25–65
Gender (*N*, % female)	23 (74%)	22 (77%)
Years since qualification (mean, range) /diagnosis of asthma (mean, range)	28.9 (1-35)	27.8 (6–65)
Number of asthma exacerbations in the past 12 months (mean, range)	n/a	2.2 (0–17)

### Adult patient with asthma interviews

Three themes captured adults’ views on introducing FeNO into asthma reviews.

#### Theme 1: Belief that FeNO will help to tailor asthma reviews to patients’ needs

Participants who felt their asthma was well controlled and who experienced infrequent or no asthma exacerbations did not see the value of attending asthma reviews as currently performed. Participants perceived these as ‘a waste of time’, as their asthma management rarely changed. They also perceived asthma reviews as ‘tick box’ exercises, felt patronised by discussions around inhaler technique, and preferred a more tailored asthma review instead of answering standard questions which may not be relevant for their asthma. Patients with severe asthma who were usually followed by a consultant or specialist in secondary care viewed primary care asthma reviews as simple ‘checking in’ opportunities, and had no expectations for their medications or asthma plan to be alter

‘*I feel like asthma reviews need to be more holistic and given the time that they deserve. I’ve always felt that mine have been quite rushed and that they did stick to the same questions every time … and I’m not sure that they used the data that’s in front of them very well, so as an example, for the last four years I’ve been going to the same GP and seeing the same asthma nurse and she’d asked me the same questions every time I would go and it was apparent that my asthma was more severe during the spring and summer months but they never once prescribed me an antihistamine or explored that further in terms of that being a trigger*’*. (Adult Patient with asthma 16)*

Participants felt that asthma reviews did not consider the individual patient, were not consistent across practices, and did not always ask tailored questions relevant to the individual. Instead, they reported being asked about their inhaler technique at every review when they were very accomplished at this already.

‘*Yeah, and I think probably it’s important to explain to people why it’s important to keep checking those things because I think sometimes, especially if you’ve had it for a long time and someone says, right, let’s go over your inhaler technique again, it can feel a bit like, oh for god’s sake, you know I know how to do this, but I think just reminding people that actually there are a lot of inhalers out there and they all have slightly different techniques. I think if people know more about the difference that having good inhaler technique can make and things like that, it can make you feel why there’s a reason for it*’*. (Adult Patient with asthma 15)*

Participants thought that introducing a test like FeNO would allow HCPs to provide more personalised asthma management allowing HCPs to make decisions based on individual factors, rather than just following standardised templates.

‘*Yeah, so it seems from what I read like it would be more personalised and very much more what I was mentioning before, where it’s more about understanding what is right and what is wrong in terms of whether it’s well managed, rather than just personal feelings*’*.(Adult Patient with asthma 18)*

#### Theme 2: Adults with asthma believed a FeNO test would help facilitate open discussions during asthma reviews

Participants reported that having an open discussion with HCPs would allow them to explain how they really feel about their asthma, discuss symptoms and expectations about medications. Participants wanted HCPs to listen to them and ask questions and felt such discussions often depended on the individual skills of the HCP.

‘*She’ll sort of say how do you feel about doing x, y, z and she’ll let you have a bit of input or she’ll sort of say well how do you want to move forwards and she will listen if I think that it is at the stage where I need a course of prednisolone or something like that. She is very good at listening and making sure you’re happy with what you’re doing moving forwards*’*. (Adult Patient with asthma 3)*

Participants thought that after doing a FeNO test HCPs would be prompted to have a more open discussion on any changes to asthma management.

‘*Yeah, I think it helps to just encourage you that you can sometimes feel like it’s in my head, am I exaggerating how I’m feeling and actually there’s something there that’s telling you actually, yes, there’s something going on here, then that I think would be helpful*’*. (Adult Patient with asthma 16)*

#### Theme 3: Adults with asthma believed that a FeNO test would increase understanding of asthma

The majority of participants were actively engaged with their asthma management, and wanted to understand their asthma better. If they had had asthma for a long time they felt they knew their own asthma better than their HCP.

‘*I think not a lot of people are used to discussing their asthma in that way, so I think a lot, what I can see for myself, is sometimes it can be a little intimidating to talk to a medical professional about your health, and especially if you have a long term condition like asthma, you can feel like it’s not seen as that important as other illnesses. It just becomes part of the everyday you just have asthma and people don’t necessarily take it that seriously so that’s how you start to think about it sometimes so when you’re in a face-to-face consultation you feel a bit like it’s difficult to sort of articulate your experiences and experience as an asthma sufferer because I guess society doesn’t necessarily see it in the way that you experience it*’*. (Adult Patient with asthma 16)*

Participants felt that a FeNO test would support them in understanding more about the state of inflammation in their lungs, how likely they are to have an asthma attack, and how they are responding to current medications. They also thought that having tangible and accessible information would increase their confidence on how well they understand their asthma, how well they can manage it, and their trust in the HCPs conducting reviews.

‘*Well, I thought it would give them a bit more information to pinpoint exactly what was going on. That’s what I thought. That’s what I got from it. So, this machine seems to tell them exactly what was going on. I would like to use it if they had one. I would love to have a go. Try it to see what’s going on now especially. If they could give me some more information with that, I think it’s a good idea. There are other things now that people can use to see what’s going on because sometimes you can’t, can you? All you can say is, I can’t breathe for some unknown reason, and that’s it*’ *(Patient 13)*

Some participants had previous experience of FeNO either in primary or secondary care. The majority of them did not have difficulties taking the test, but many reported that they did not receive enough information before conducting the test. They wanted to know what to expect from a new test. They wanted to be informed in a simple way about benefits, practical aspects of the test, and about test results. One patient, who had a separate FeNO test appointment prior to their routine review, had their asthma review and discussion of result over the phone afterwards to minimise face-to-face contact during the Covid pandemic. They reported a lack of communication of what their test result meant, leaving them with worries about potential implications for their asthma management. This suggested that adults with asthma want an explanation of a how a FeNO test result would affect decisions about treatment.

‘*She said she couldn’t tell me whether that was good or bad, to which I said well you might as well tell me because I’m only going to go home and Google it anyway, which I did. So I think if I was the sort of person who was worried about this thing and I’d had the test and got something that was three or four times more than perhaps it should be and I went home and Googled it, I have been a bit concerned if I wasn’t a less concerned type of person, would be my comment*’*. (Adult Patient with asthma 22)*

### Health Care Professional interviews

Two themes captured HCPs’ views on the use of FeNO in routine asthma reviews.

#### Theme 1: FeNO as an objective measurement of inflammation that could inform tailored asthma management

Some HCPs (mainly GPs), reported that it was useful to identify patients based on their asthma symptom control to identify those who would benefit most from a face-to-face review. Participants felt that those patients with less well-controlled asthma would benefit more than those with well-controlled asthma. In addition, participants believed that well-controlled patients mainly see reviews as ‘tick-box exercises’ and a ‘waste of time’, and reported that their perception was that patients had responded more positively to recent changes to asthma reviews being conducted over the telephone.

‘*Sometimes people don’t like talking about themselves and what they’re doing, they don’t want to admit that they’re smoking, so they don’t want someone being intrusive. I think some people just think their asthma’s well-controlled and it’s a waste of their time to be contacted to be reviewed*’*. (Practice Nurse)*

HCPs felt that some patients get used to their symptoms and mistakenly consider themselves as having well-controlled asthma, highlighting the need for more objective measures to assess inflammation, other than relying on patient evaluations. HCPs reported that the introduction of the FeNO test would provide information on inflammation which ultimately would allow them to provide more informed treatment.

‘*Actually seeing how the inflammation affects people and equally then seeing how their medication affects that inflammation I can see the benefits of [FeNO] being a more tailored approach*’*. (Practice Nurse)*

When considering ways to implement the FeNO test, HCPs reported being more willing to try a new test if it fitted into the existing consultation, templates, and electronic systems. GPs reported having limited time to do asthma reviews, whereas nurses and pharmacists were considered better placed, and were willing, to use the test during reviews. The main barrier to implementation that HCPs reported was the cost of the analyser, patient filters, and maintenance of analysers. HCPs also wanted appropriate training about factors that could affect the test results, interpreting correctly test results, and caring for the analyser.

‘*When we’re doing [FeNO] in a consultation will it add a lot of time burden onto the consultation? I personally don’t think so. […] So, in that time slot that I have, if I was to say for every patient I have to do a FeNO now, will that make a difference? Not really. Not in terms of the management, I mean by the administrative side. I don’t think it would put a time strain in terms of me rushing. Yeah, I think it would just be part of the new process*’*. (Pharmacist)*

#### Theme 2: Use of FeNO to educate and empower patients

HCPs reported that an important aspect of asthma reviews is to educate patients about asthma and inhaler techniques. They reported often using external resources, such as videos and leaflets from asthma charities such as Asthma UK, to support medication adherence.

‘*I feel that where we can try and improve patient education, patient-based understanding of their treatment will obviously help with adherence to their treatment and help with their overall management of asthma*’*. (Pharmacist)*

All HCPs believed that having a patient-centred, holistic approach was best when discussing treatment and asthma management during reviews. They reported doing this to increase patient empowerment and ownership in management decisions, which was believed to increase both medication adherence and patient satisfaction.

‘*I think what I’ve tried to develop over the time I’ve been doing it is trying to give patients control over their condition and trying to allow them to understand that whilst there are specific things we can or can’t prescribe, we endeavour to make them suit them and fit their needs. We’re often under a lot of pressure to use certain inhalers and now obviously there’s a big, big push on dry powder inhalers, but there’s certain patients that that’s just not suitable, so for me it’s trying to develop that relationship and trying to understand the patient’s specific needs*’*. (Practice Nurse)*

HCPs reported that when introducing a new test it also needed to be easy to explain to patients. For this reason, they liked that the FeNO test was a simple number, seen as easy to interpret and communicate to patients. HCPs believed that patients would like the test and would accept it as part of usual asthma care, viewing it as easier compared to other tests currently used.

‘*I think the device looked quite nice and easy and quite user friendly, because I think often if we get given a new bit of equipment, sometimes it’s easier to be fearful and think oh my goodness, how does this work? If it’s complicated I think it puts us all off because I think sometimes when everything is really busy, you just think oh gosh no, please not something that’s going to be tricky, so it did look quite nice and straight forward. I think if you’ve got a patient in here already then I think it seemed relatively easy to do, quite painless and it looked quite quick*’*. (Practice Nurse)*

Health care professionals reported the importance of education and communication about decisions on medications, and reported challenges regarding discussions about reducing medications. This highlighted the importance of providing convincing explanations to patients about how their FeNO result informed asthma management and the safety of subsequent treatment decisions.

‘*The most difficult thing, I think, is trying to convince a small number of people*—*those who use Ventolin as a habit*—*convincing them not to take it. But, on the whole, I think patients will accept changes in medication if you explain why you do it*’*. (Practice Nurse)*

‘*I find them quite receptive, because if I’ve explained it to them clearly and they understand, then they know they’re going to be feeling better. So, they’re usually pretty up for it, even if it’s a change of device. Pretty good. Quite receptive to it, yeah*’*. (Practice Nurse)*

## Discussion

This study explored patients’ and health care professionals’ views on the adoption and use of FeNO within their existing experience of primary care asthma reviews. It has highlighted key barriers and facilitators to consider when introducing FeNO testing for the first time in primary care.

Adults with asthma reported wanting asthma reviews to be more tailored to their needs. Similar to previous qualitative research on collaborative care and communication during asthma reviews^11,19^, adults with asthma wanted HCPs to have an holistic approach and to communicate openly about patient symptoms, the impact of their asthma on their everyday life, and about asthma management. They also believed that having an objective and tangible measure of airways inflammation, such as the FeNO test, would allow HCPs to provide a more personalised review, rather than having a ‘tick box’ review, like the ones they often had in the past. All adults with asthma were happy to conduct the FeNO test as it was seen as something easy to do, compared to existing tests.

Health care professionals had mixed views about introducing FeNO into routine asthma reviews. The majority of HCPs reported that the key benefit of doing a FeNO test would be to have an objective measure of airway inflammation in order to provide better patient care and educate patients on risks of asthma attacks. This supports the finding of a qualitative study on clinicians’ views on practices, tests and challenges when diagnosing asthma, on the need for new models of care to provide cost-effective access to accurate and potentially novel tests^20^. The new models of care have previously been identified as patient-centred personalised asthma management^21^. The health care professionals we interviewed also thought that having the FeNO test result would allow them to assess patients’ asthma more comprehensively rather than relying on reported measurement of symptoms. Previous studies also reported that asthma symptoms correlate weakly with the level of airway inflammation^22^.

Analysis reported some differences between sub-groups of HCPs: nurses were especially willing to use FeNO if it fitted into the existing consultation, templates, and electronic systems.

However, although the test was perceived as quick and easy to administer, the main barriers to implementation that HCPs reported were the cost of the analyser, patient filters and maintenance of analysers, and lack of knowledge about factors that could affect the test results, confidence in how to correctly interpret test results, and how to care for the FeNO analyser.

This study explored both adults with asthma and clinicians’ views on the adoption and use of FeNO in primary care asthma reviews. It has highlighted key barriers and facilitators to the implementation of FeNO. This study fills an important gap in literature providing adults with asthma and clinicians’ perspectives.

Despite participants being recruited using a purposive sampling strategy, the sample of adults with asthma was not be representative of the UK population of patients with asthma, as they had to fit certain eligibility criteria. More specifically, we did not interview any patients from ethnic minority groups, or younger patients with asthma (under 16 years old). In addition to this, the participants recruited may have been more interested in FeNO than those who did not volunteer, highlighting that we may have not succeeded in sampling HCPs and patients who are less receptive to the use of FeNO.

The COVID-19 pandemic may have had an impact on interview participants’ views on the general importance and logistics of conducting routine asthma reviews, as many GP surgeries either delayed such reviews or opted to conduct them remotely where possible when the pandemic started. However, our data do not suggest that the pandemic affected participants’ views on FeNO testing, since it was accepted that if this was to be done as part of an asthma review, it would have to be done face to face, irrespective of the pandemic. Moreover, due to travel restrictions we did have to conduct interviews over the phone and online (using MS teams) based solely on participants’ choice over the two methods of interviewing. We believed that both types of interviews allowed us to explore in depth participants’ views on the use of FeNO on asthma reviews and were similar in nature, although when online video reviews were conducted the interviewer could have relied also on non-verbal cues.

Finally, the findings of this study need to be considered carefully in terms of transferability to other countries as we recruited from practices in England.

FeNO is seen as a potentially acceptable test to be used in primary care asthma reviews by both adults with asthma and clinicians, as it is seen as easy to perform and as providing objective measure of airways inflammation, which could provide more personalised treatment. Health care professionals need to be supported when implementing the test, with appropriate training about factors that could affect the test results, interpreting test results correctly, and open communication about test results with patients in the context of their asthma.

## Supplementary information

Reporting Summary

## Data Availability

The data that support the findings of this study are available from the corresponding author upon reasonable request.
