# Supplementary material for: Qualitative study on perceptions of use of Fractional Exhaled Nitric Oxide (FeNO) in asthma reviews
Source: NPJ Prim Care Respir Med. 2022 Mar 21;32:13. doi: 10.1038/s41533-022-00272-0 (PMC8938430; doi:10.1038/s41533-022-00272-0)
Supplement: Supplementary file 1 — Reporting Summary [file 41533_2022_272_MOESM1_ESM.pdf]

nature portfolio

Corresponding author(s): Dr Marta Santillo
Last updated by author(s): Oct 21, 2021

Reporting Summary

Nature Portfolio wishes to improve the reproducibility of the work that we publish. This form provides structure for consistency and transparency in reporting. For further information on Nature Portfolio policies, see our Editorial Policies and the Editorial Policy Checklist.

Statistics

For all statistical analyses, confirm that the following items are present in the figure legend, table legend, main text, or Methods section.

n/a	Confirmed	
☒	☐	The exact sample size (*n*) for each experimental group/condition, given as a discrete number and unit of measurement
☒	☐	A statement on whether measurements were taken from distinct samples or whether the same sample was measured repeatedly
☒	☐	The statistical test(s) used AND whether they are one- or two-sided<br>*Only common tests should be described solely by name; describe more complex techniques in the Methods section.*
☒	☐	A description of all covariates tested
☒	☐	A description of any assumptions or corrections, such as tests of normality and adjustment for multiple comparisons
☒	☐	A full description of the statistical parameters including central tendency (e.g. means) or other basic estimates (e.g. regression coefficient) AND variation (e.g. standard deviation) or associated estimates of uncertainty (e.g. confidence intervals)
☒	☐	For null hypothesis testing, the test statistic (e.g. *F*, *t*, *r*) with confidence intervals, effect sizes, degrees of freedom and *P* value noted<br>*Give P values as exact values whenever suitable.*
☒	☐	For Bayesian analysis, information on the choice of priors and Markov chain Monte Carlo settings
☒	☐	For hierarchical and complex designs, identification of the appropriate level for tests and full reporting of outcomes
☒	☐	Estimates of effect sizes (e.g. Cohen's *d*, Pearson's *r*), indicating how they were calculated

Our web collection on statistics for biologists contains articles on many of the points above.

Software and code

Policy information about availability of computer code

Data collection — *Provide a description of all commercial, open source and custom code used to collect the data in this study, specifying the version used OR state that no software was used.*

Data analysis — *Provide a description of all commercial, open source and custom code used to analyse the data in this study, specifying the version used OR state that no software was used.*

For manuscripts utilizing custom algorithms or software that are central to the research but not yet described in published literature, software must be made available to editors and reviewers. We strongly encourage code deposition in a community repository (e.g. GitHub). See the Nature Portfolio guidelines for submitting code & software for further information.

Data

Policy information about availability of data

All manuscripts must include a data availability statement. This statement should provide the following information, where applicable:

- Accession codes, unique identifiers, or web links for publicly available datasets
- A description of any restrictions on data availability
- For clinical datasets or third party data, please ensure that the statement adheres to our policy

Provide your data availability statement here.

Field-specific reporting

Please select the one below that is the best fit for your research. If you are not sure, read the appropriate sections before making your selection.

☐ Life sciences ☒ Behavioural & social sciences ☐ Ecological, evolutionary & environmental sciences

For a reference copy of the document with all sections, see nature.com/documents/nr-reporting-summary-flat.pdf

Behavioural & social sciences study design

All studies must disclose on these points even when the disclosure is negative.

Study description	The main aim of the study is to find out healthcare professionals' and patients' views and experiences of FeNO-guided asthma management and their views on information materials about FeNO. We conducted semi-structured interviews with people with asthma and health care professionals (HCPs) who perform asthma review consultations in primary care.
Research sample	Health care professionals (e.g. doctors, nurses, pharmacists) who manage patients with asthma in primary care. Maximum variation sample based on job role, years of experience, previous additional training in respiratory medicine or asthma management, practice list size and practice deprivation.<br>People/patients diagnosed with asthma. Maximum variation sample based on age, gender, time since diagnosis of asthma and history of previous exacerbations.
Sampling strategy	We will seek to obtain maximum variation sampling for all participant groups. Health care professionals taking part in semi-structured interviews and audio- or video recordings of asthma review consultations will be sampled based on job role (doctor, nurse, pharmacist, other health care professional), years of experience, previous additional training in respiratory medicine or asthma management. Where possible health care professionals will be sampled from practices that vary in patient list size and socioeconomic deprivation (as assessed by Index of Multiple Deprivation score according to practice postcode). Variation in these factors will allow us to identify the needs and experiences of health care professionals with different training (medical, nursing, pharmacy) and levels of experience to assess how to support materials to promote the use of FeNO can be best designed to help these professionals.<br>Patients/people with asthma taking part in interviews will be sampled based on age, sex, time since diagnosis of asthma and history of previous exacerbations. Variation in these factors should help explore varied experiences of asthma and identify how materials to explain the use of FeNO and subsequent management of asthma can be best written to be understandable and useful to patients.
Data collection	HCPs and patients will participate in semi-structured interviews following a topic guide (one for each participant group,). All interviews will include a "think-aloud" component where HCPs and patients will look through intervention materials, supporting use of FeNO in primary care, and comment on the design, format and content. Interviews may be carried out remotely by telephone or online (e.g. Microsoft Teams or Skype). Interviews will be carried out according to participant preferences and to cause least disruption to participants. Interviews will be audio recorded and field notes will also be taken.
Timing	July 2020-January 2021
Data exclusions	no data was excluded
Non-participation	no participants dropped out
Randomization	n/a

Reporting for specific materials, systems and methods

We require information from authors about some types of materials, experimental systems and methods used in many studies. Here, indicate whether each material, system or method listed is relevant to your study. If you are not sure if a list item applies to your research, read the appropriate section before selecting a response.

Materials & experimental systems

n/a	Involved in the study	
☒	☐	Antibodies
☒	☐	Eukaryotic cell lines
☒	☐	Palaeontology and archaeology
☒	☐	Animals and other organisms
☐	☒	Human research participants
☒	☐	Clinical data
☒	☐	Dual use research of concern

Methods

n/a	Involved in the study	
☒	☐	ChIP-seq
☒	☐	Flow cytometry
☒	☐	MRI-based neuroimaging

Human research participants

Policy information about studies involving human research participants

Population characteristics	see above
Recruitment	Different strategies were employed to recruit participants. To recruit healthcare professionals, we sought support from Thames Valley and South Midlands Clinical Research Network (CRN) to invite general practices and their staff. To be eligible to participate primary care health care professionals (including general practitioners, nurse practitioners and pharmacists) had to carry out routine asthma reviews with patients and, where qualified, make decisions about asthma medication. We sought to obtain a maximum variation sample based on job role, years of experience, additional training in respiratory medicine or asthma management, practice list size. Practices expressed their interest in taking part in the study directly to the CRN which then informed the research team by email. Health care professionals eligible to take part were then purposively selected and contacted to arrange an interview.<br>To recruit adults with asthma, we sent invitations through general practices, advertised through Asthma UK and through Twitter and Facebook. Participants with asthma were invited to contact the research team or let their practice know if they were interested in taking part. To be eligible, people had to be diagnosed with asthma, fluent in English, aged 12 or over, or a parent/carer of a child aged 12-15 diagnosed with asthma. Maximum variation sampling was based on age, gender, time since diagnosis of asthma and history of previous exacerbations, in order to capture a range of disease severity and control, as we felt these would be key in influencing patients' perceptions of FeNO. Adults with asthma who expressed interest were contacted by email to arrange an interview.
Ethics oversight	NHS REC ethics approval (South Central - Berkshire B Research Ethics Committee, 20/SC/0235).

Note that full information on the approval of the study protocol must also be provided in the manuscript.
